# Supplementary material for: Acoustic phonon recycling for photocarrier generation in graphene-WS2 heterostructures
Source: Nat Commun. 2020 Aug 3;11:3876. doi: 10.1038/s41467-020-17728-x (PMC7400626; doi:10.1038/s41467-020-17728-x)
Supplement: Supplementary file 1 — Supplementary Information [file 41467_2020_17728_MOESM1_ESM.pdf]

Supplementary Information

Acoustic phonon recycling for photocarrier generation in graphene-WS_2 heterostructures

Wei *et al.*

Supplementary Figures

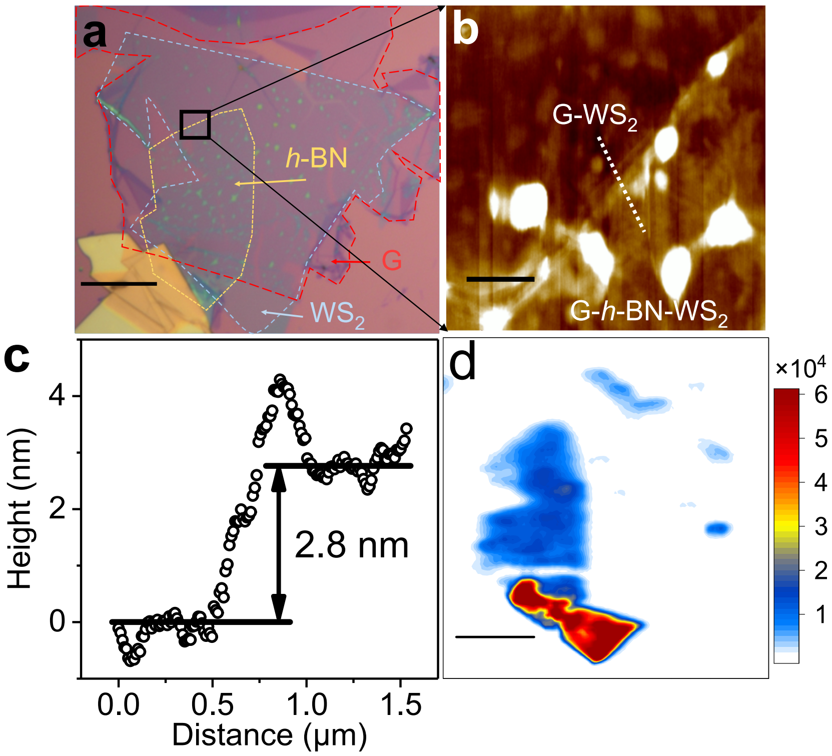

Supplementary Figure 1: Optical characterization of Sample 1. (**a**) Optical image. Scale bar, 10 μm. (**b-c**) AFM height image and height profile, showing the 4~5 layer thickness (2.8 nm) of the *h*-BN spacer. Scale bar in (b), 1 μm. (**d**) Integrated PL intensity image showing the coupling strength of the heterostructure. Scale bar, 10 μm.

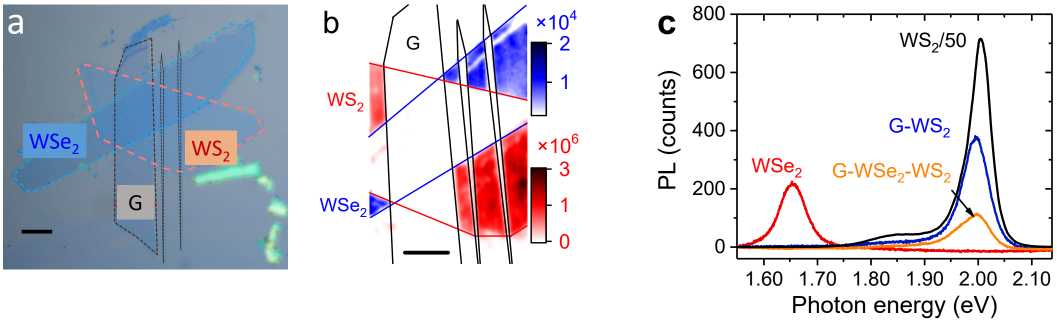

Supplementary Figure 2: Optical characterization of Sample 2. (**a**) Optical image. Scale bar, 5 μm. (**b**) Integrated PL intensity image from the peaks of WS_2 (615~625 nm, red) and WSe_2 (745~755 nm, blue). Scale bar, 5 μm. (**c**) PL spectra in different regions. For comparison, the PL in WS_2 is scaled down by 50 times, and PL in monolayer WSe_2 is also plotted.

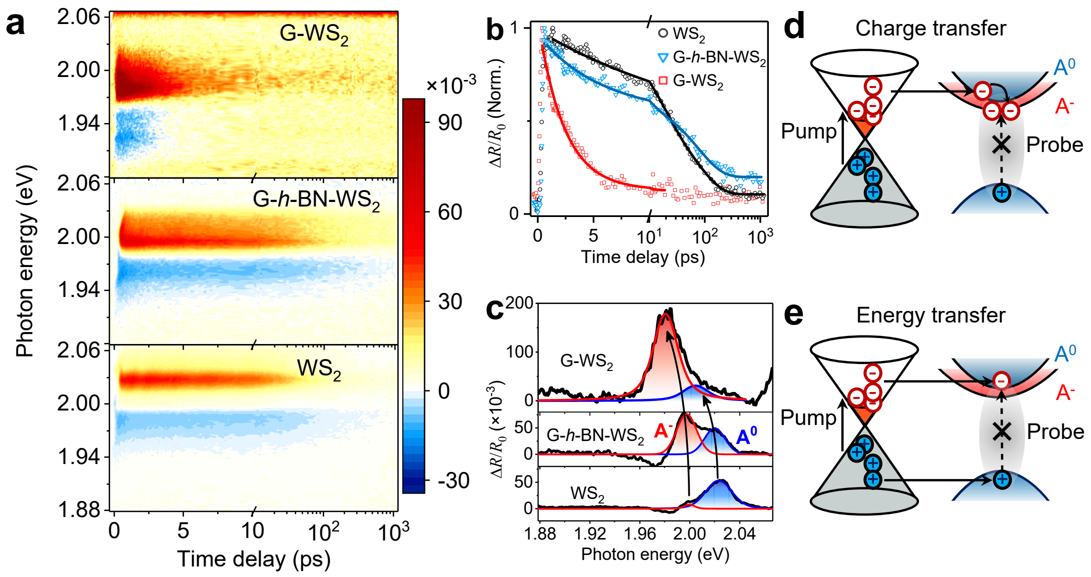

Supplementary Figure 3: TA measurements under up-bandgap pumping (~2.1 eV, 7.8 μJ cm^{-2}). (**a**) TA image of the three regions in Sample 1 and (**b**) the corresponding normalized exciton relaxation dynamics. (**c**) TA spectra fitted with two components, namely, neutral exciton (blue, A^0) and charge trion (red, A^-). (**d**) and (**e**) Illustration of the charge and energy transfer-induced PB peaks in the TA spectrum. Charge transfer leads to net charge carriers in WS_2, which may further combine with the excitons and become trions. These trions occupy the energy state and reduce the absorption of the probe, forming a PB peak at the trion resonance. While the energy transfer leaves neutral exciton in WS_2, and thus a PB feature occurs at the exciton resonance.

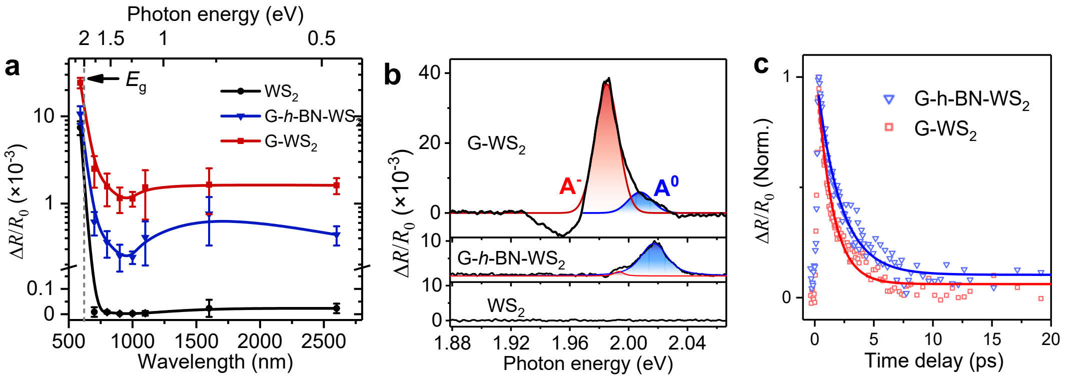

Supplementary Figure 4: TA signals induced by electronic excitation from energy/charge transfer. (**a**) TA response per unit pump fluence (μJ cm^{-2}) at different pump energy (2.1~0.48 eV). The error bars correspond to the s.d. measured from 5 different areas. (**b-c**) Typical subbandgap-pumping TA spectra (b) and TA dynamics (c) measured at different sample regions, with a pump energy of 1.38 eV (900 nm) and pump fluence of ~34 μJ cm^{-2}.

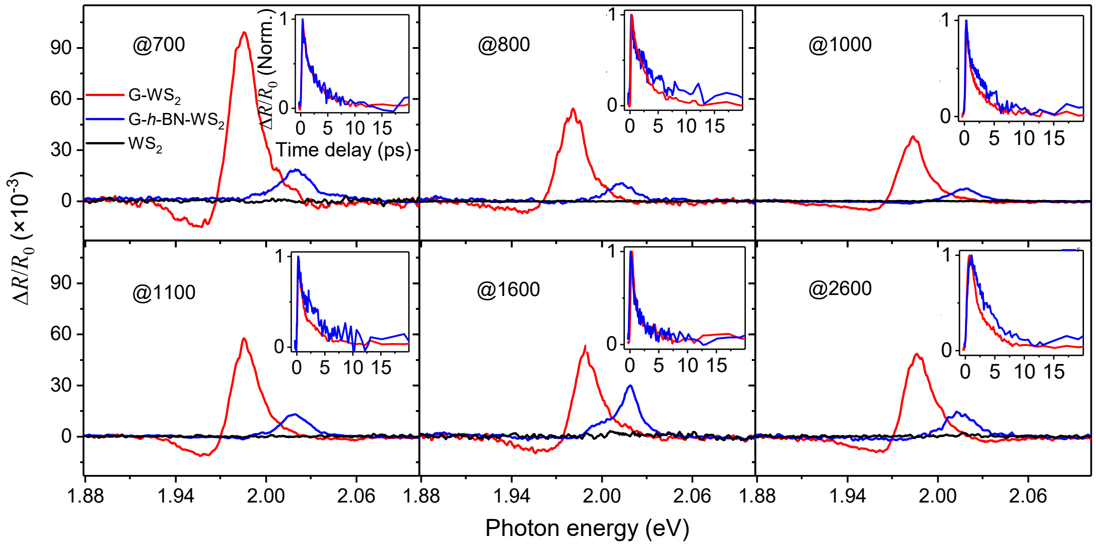

Supplementary Figure 5: Short time scale TA spectra (~0.5 ps, main panel) and TA kinetics (normalized, inset) measured from different sample regions (WS_2, G-WS_2, and G-*h*-BN-WS_2), with the pump energy changing from 700 nm to 2600 nm. For all the pump energies, monolayer WS_2 shows no detectable signal, while G-WS_2 (G-*h*-BN-WS_2) exhibits a PB feature dominated by A^- (A^0) peak, indicating charge (energy) transfer from G to WS_2. The transferred carriers/excitons show a similar decay dynamic, with an ultrafast lifetime of 1~2 ps, indicating that these carriers/excitons mainly decay through nonradiative recombination channel.

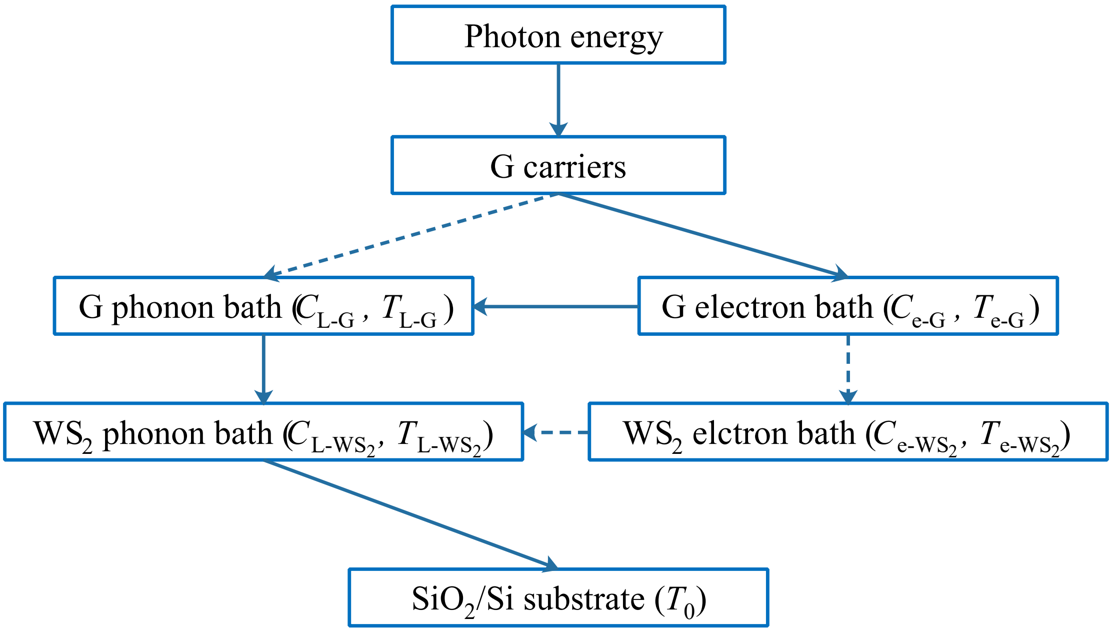

Supplementary Figure 6: Schematics illustrating the cooling pathways of the photo-excited carriers in a G-WS_2 heterostructure on SiO_2/Si substrate. Detail discussions can be found in Supplementary note 3.

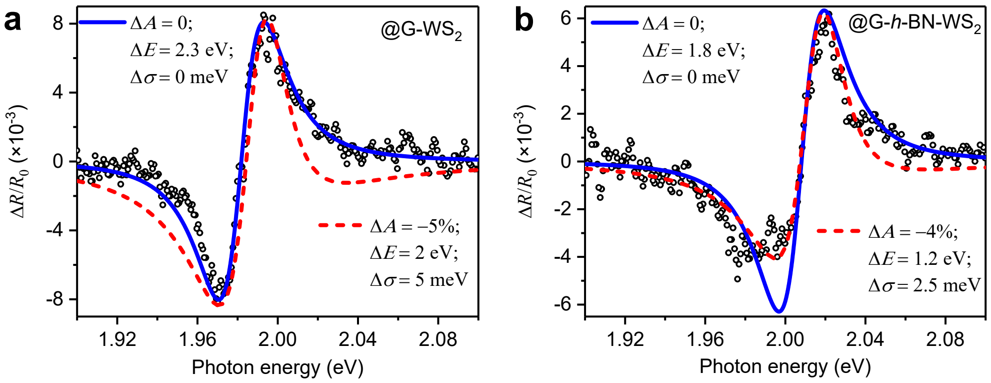

Supplementary Figure 7: Long time scale (≥ 100 ps) TA spectra of the (a) G-WS_2 and (b) G-*h*-BN-WS_2 heterostructures. Dots are experimental results, and blue lines and red dashes are fittings with and without the band filling effect (ΔA).

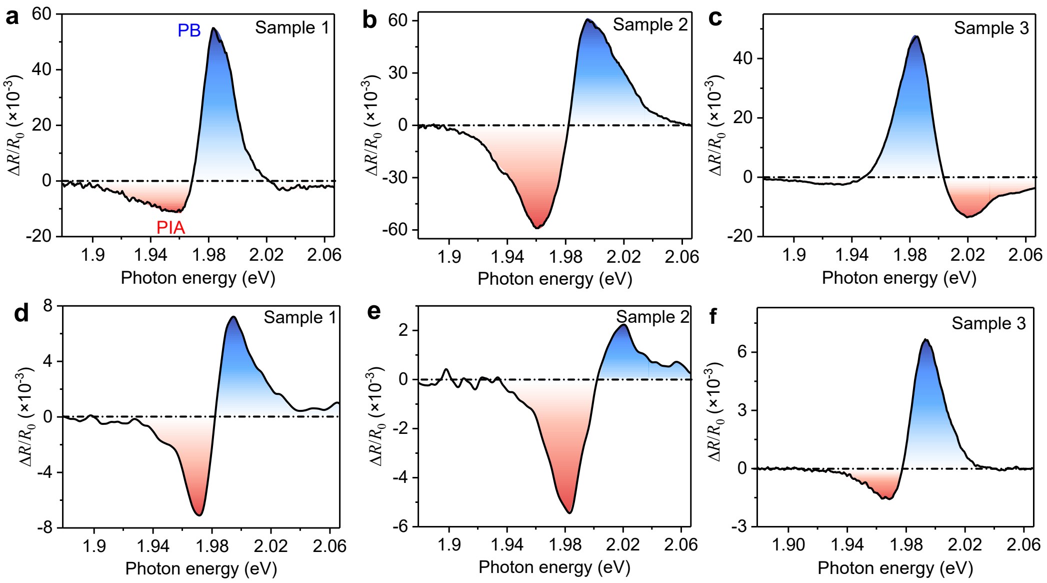

Supplementary Figure 8: Transition absorption spectra in different samples. (**a-c**) TA spectra induced by electronic excitation in Sample 1~3. (**d-f**) TA spectra induced by phononic excitation in Sample 1~3. The PB and PIA feature are marked by the blue and red areas. Note that both the amplitude and energy of the PIA feature (red area) vary from sample to sample compared to the PB peak (blue area). The optical image and detailed TA data of Sample 3 can be found in Supplementary Figure 11 (φ = 1.5°).

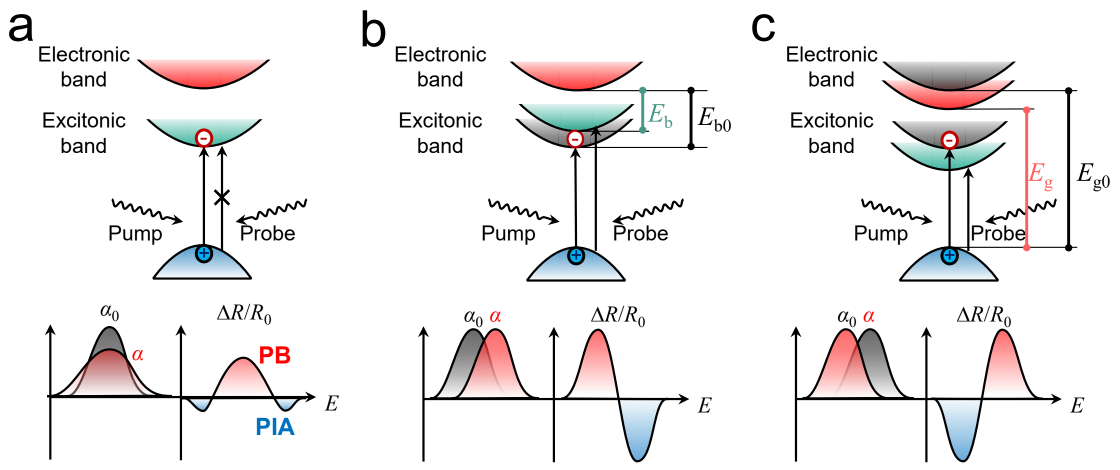

Supplementary Figure 9: Schematics showing TA spectrum induced by different kinds of many-body effects. (**a**) Phase-space filling and Coulomb scattering induce a photo bleaching and spectral broadening. (**b**) Screening of the Coulomb attraction induces a decrease of exciton binding energy (E_b). (**c**) Screening of the Coulomb repulsion induces a decrease of electronic bandgap (E_g). The up panel illuminates the many-body effects and the below panel shows corresponding TA spectra.

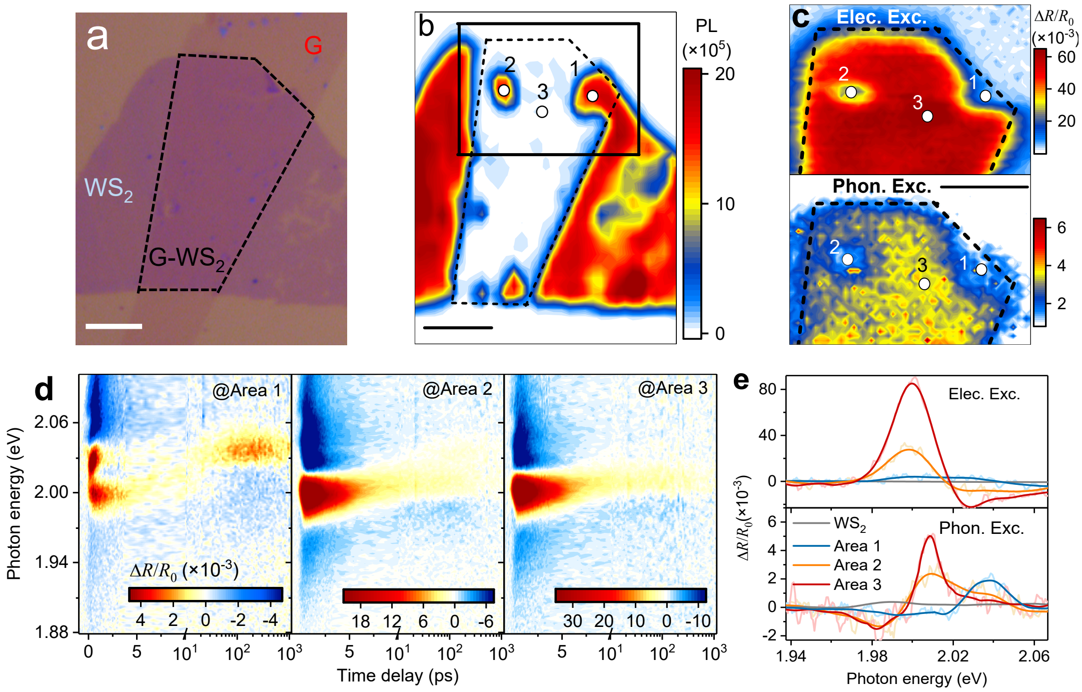

Supplementary Figure 10: Interfacial coupling-dependent APR effect. (**a**) Optical image of a non-annealed G-WS_2 sample (Sample 4). The heterostructure is marked by the dashed box. (**b**) PL intensity image showing the coexistence of well and terrible coupling regions. (**c**) TA intensity images measured at time delay of 0.5 ps (up panel) and 100 ps (below panel), with the mapping area shown in (b), solid rectangle. Scale bar in (a-c), 5 μm. (**d**) 2D pseudo-color TA maps of the three heterojunction areas marked in (b). (**e**) TA spectra of the three heterojunction areas marked in (b), with time delays of 0.5 ps (up panel) and 100 ps (below panel).

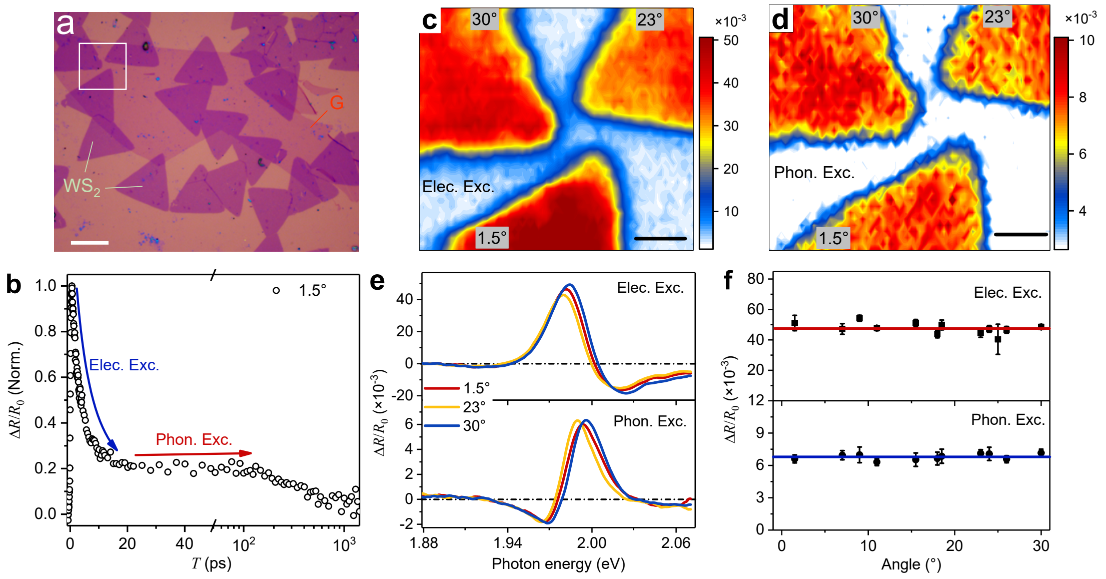

Supplementary Figure 11: Stacking-independent APR effect. (**a**) Optical image of a typical G-WS_2 sample with different stacking configurations (Sample 3). Scale bar, 20 μm. (**b**) Carrier relaxation dynamics in a G-WS_2 heterostructure with $\varphi = 1.5°$. (**c-d**) TA intensity images induced by electronic (c) and phononic (d) excitations, measured at delays of 0.5 ps and 100 ps, respectively. Scale bar, 5 μm. (**e**) TA spectra caused by electronic (up panel) and phononic (below panel) excitations from three stacking configurations ($\varphi = 1.5°$, 23° and 30°). (**f**) Electronic (up panel) and phononic (below panel) excitation intensities versus twist angle between graphene and WS_2. The error bars correspond to the s.d. measured from 10 different areas. No correlation is found between these two excitations and twist angle.

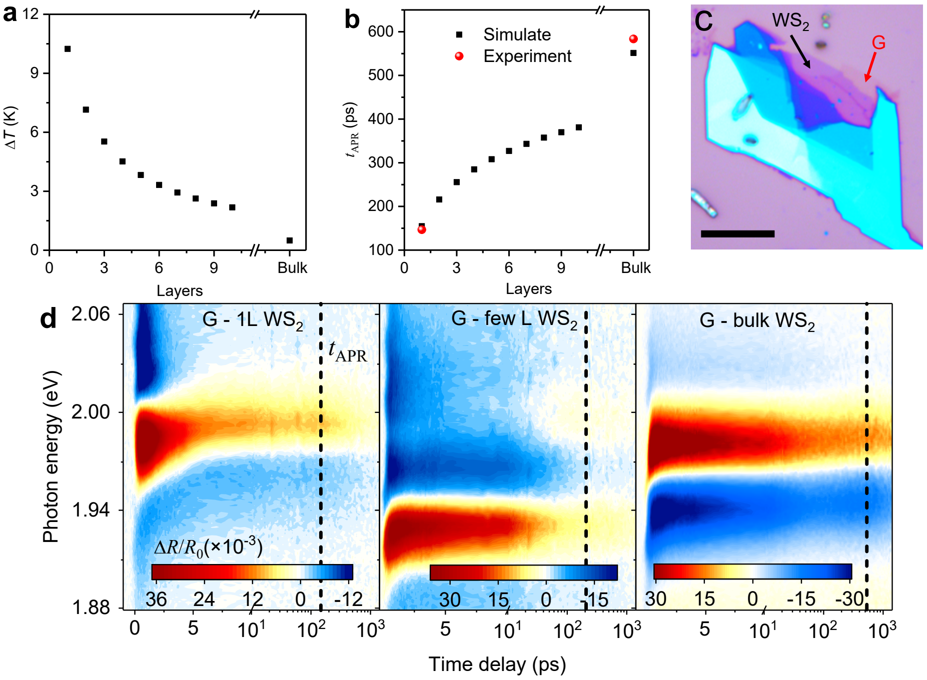

Supplementary Figure 12: APR effect in G-WS_2 with different WS_2 thicknesses. (a-b) Simulated WS_2 layer dependent maximum temperature increase (a) and APR characteristic time (b) under a pump fluence of ~34 μJ cm^{-2}. For comparison, Experimental results of the t_{APR} is also plotted at (b), red circles. (**c**) Optical image of a typical G - nL WS_2 sample (Sample 5). Scale bar, 10 μm.(**d**) 2D pseudo-color TA maps of different heterostructure regions, including G-1L WS_2, G-few L WS_2 and G-bulk WS_2, the APR characteristic time is marked by the dash line.

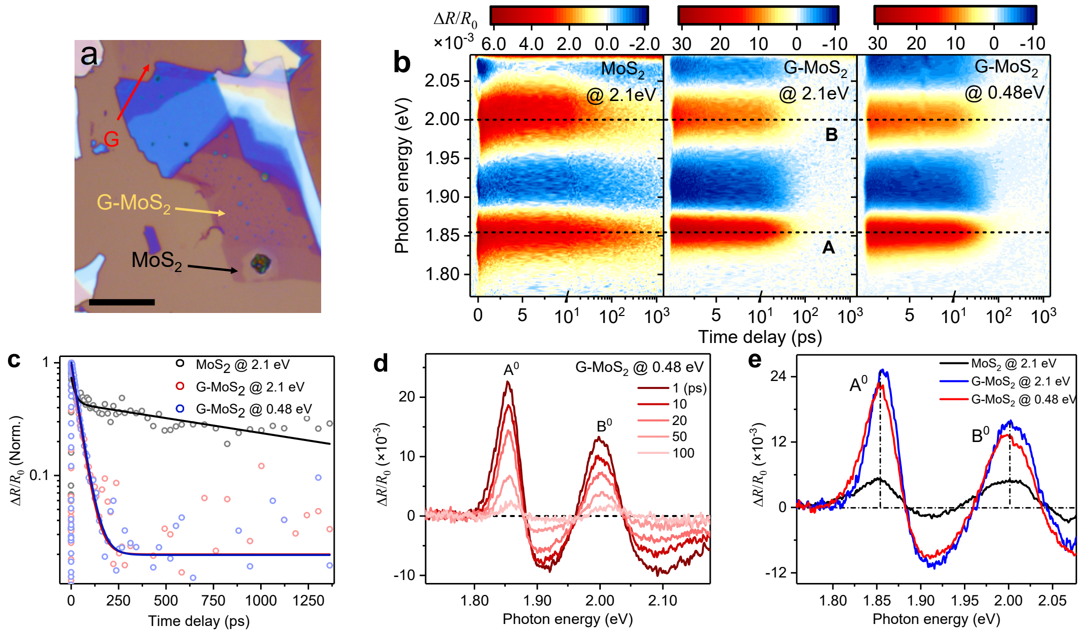

Supplementary Figure 13: APR effect in an as-prepared G-MoS_2 sample. (**a**) Optical image of a typical G-MoS_2 sample (Sample 6). Scale bar, 10 μm. (**b**) 2D pseudo-color TA maps of G-MoS_2 heterostructure under different pump energy and pump regions. Left, controlled MoS_2, E_{pump} = 2.1 eV, middle, G-MoS_2, E_{pump} = 2.1 eV and right, G-MoS_2, E_{pump} = 0.48 eV. (**c**) Corresponding carrier relaxation dynamics. (**d**) TA spectra of the G-MoS_2 at different time delays, with pump photon energy of 0.48 eV. (**e**) The comparison of TA spectra from different sample regions. All the TA spectra are dominated by neutral excitons (A^0 and B^0), indicating that the Fermi energy locates at a level near the center of the bandgap.

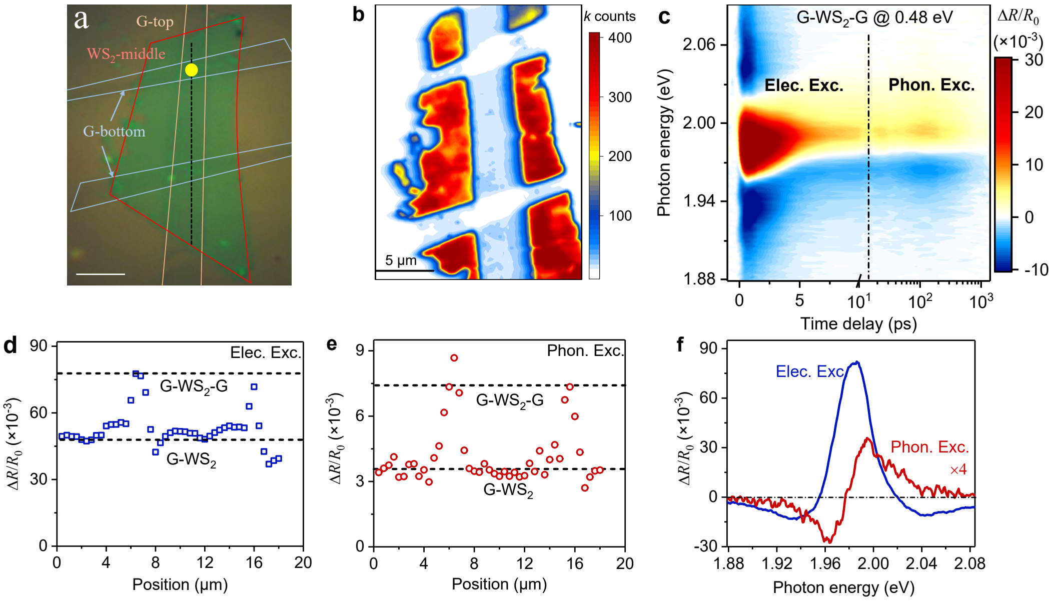

Supplementary Figure 14: APR effect in the G-WS_2-G heterostructure. (**a**) Optical image of a G-WS_2-G sample (Sample 7). Scale bar, 5μm. (**b**) PL intensity mapping showing the well coupling of the heterostructures. Scale bar, 5μm. (**c**) 2D pseudo-color TA maps measured at the G-WS_2-G region marked in (a), yellow point. (**d-e**) Electronic (d) and phononic (e) excitation intensity mapping along the line shown in (a), black dash. (**f**) A comparison of the TA spectra induced by the electronic and phononic excitations.

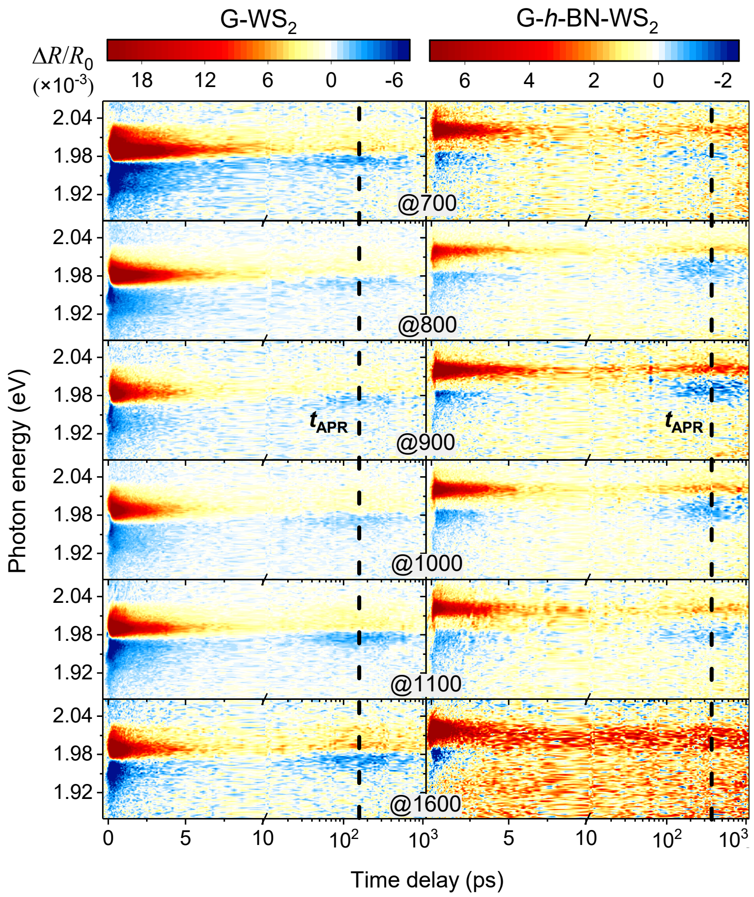

Supplementary Figure 15: TA image at different pumping energies (i.e., 700-1600 nm). The left (right) panel is measured at G-WS_2 (G-*h*-BN-WS_2) region in Sample 1. Both electronic and phononic excitations are found in all pump energy and regions. The phononic characteristic time (t_{APR}) is ~150 ps (~400 ps) in G-WS_2 (G-*h*-BN-WS_2) region, which is independent of the pump photon energy.

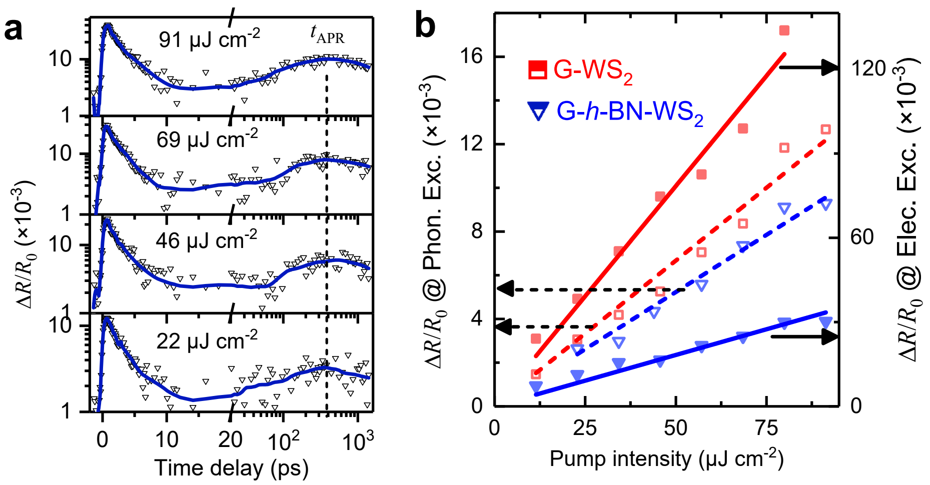

Supplementary Figure 16: Pump fluence-dependent APR process. (**a**) Differential reflectance kinetics measured in heterostructure with *h*-BN spacer (G-*h*-BN-WS_2), accounting for different pump intensities. Dots are measurement results and solid lines are smooth data for clarify. (**b**) The peaks of the signal induced by electronic (right axis, solid) and phononic (left axis, dash) excitations as a function of pump fluence. For comparison, data measured from G-WS_2 (red) and G-*h*-BN-WS_2 (blue) regions are both shown. Dots are measurement results, and lines are fitting from APR model.

Supplementary Note 1: Sample characterization

Two kinds of samples are used in the main text, the first one (Sample 1) is shown in Supplementary Figure 1, which consists of three different regions, namely, the monolayer WS_2, G-WS_2 and G-*h*-BN-WS_2. The *h*-BN thickness is confirmed to be 4~5 layers by the atomic force microscope (AFM) (Supplementary Figure 1(b) and (c)). The selection of this specific *h*-BN thickness serves as two purposes: firstly, for the ultrafast interfacial carrier transfer (<10 ps), *h*-BN is an insulator that can block a part of carrier transfer (i.e. charge transfer, see Note 2). Secondly, for the interfacial thermal transport, *h*-BN is an excellent thermal conductor which can efficiently transfer the heat from graphene to WS_2 for carrier re-excitation. In comparison, completely opposite property (electrical conductor and thermal insulator) is exhibited in the monolayer WSe_2 spacer, which is inserted in Sample 2(Supplementary Figure 2). These two different samples allow one to comparatively study the acoustic phonon recycling (APR) effect.

In both samples, the PL intensity in G-WS_2 region quenches ~100 times compared to that of monolayer WS_2, indicating well interlayer coupling of the heterostructures. The PL quenching amplitude is even higher (>600 times) in G-WSe_2-WS_2 region, since WSe_2 is an electric conductor that cannot block the interfacial carrier transfer. Only ~4 times PL quenching is found in G-*h*-BN-WS_2 region owing to the electric insulating property of the *h*-BN spacer. The small PL quenching in G-*h*-BN-WS_2 region is attributed to the impurity doping-induced trion emission (discussed in the text).

Supplementary Note 2: Charge and energy transfers in the ultrafast electronic excitation region

Although massive studies[1-4] have investigated the ultrafast carrier transfer in G-TMDCs heterostructures, it is still not clear whether charge or energy transfer dominates this interfacial process. Clarification of this issue is of particular importance, since these two types of carrier transfer dictate different applications. Specifically, charge transfer is beneficial to the light-conversion or detection devices while energy transfer is often used in light-emitting devices. Here, we show a possible approach to distinguish the charge and energy transfers by using the spectrally resolved transient absorption microscopy.

Up-bandgap pumping case is firstly used to directly excite the WS_2 carriers and study the carrier transfer from WS_2 to graphene. To avoid complications (such as hot carriers thermalization and intraband carrier cooling) that may obfuscate analysis and interpretation[5], we use a nearly resonant energy (590 nm, 7.8μJ cm^{-2}) to pump the heterostructure. Supplementary Figure 3(a) shows the pseudo-color maps of TA signals measured at the three regions in Sample 1. All the signals show strong photo-bleaching (PB) peaks with the same energy as the PL emissions (see the main text), which is attributed to the phase-space filling of the photo excitations (either A^0 or A^-). The relaxation dynamics of the excitons are presented in Supplementary Figure 3(b). The monolayer WS_2 and G-*h*-BN-WS_2 regions show roughly the same biexponential decay, with the fast components of 14 ps (50%) and 5 ps (45%) and the same slow component of 77 ps (50% and 55%), respectively. This similar kinetics indicates that a 4~5 layer *h*-BN (2.8 nm) is thick enough to block the interfacial charge transfer. In comparison, the excitons in G-WS_2 region decay much faster due to the efficiently interfacial carrier transfer, with a lifetime of ~2.4 ps, which is well consistent with

previous reports[2].

To show whether the charge or energy transfer dominates this interfacial process, we carefully look up into the TA spectra of the three regions in Sample 1, as shown in Supplementary Figure 3(c). The TA spectra can be fitted well with two Gaussian peaks, A^0 and A^-, with an energy difference of ~25 meV (A^- binding energy)[6]. This means that we might be able distinguish the energy and charge transfers by checking the relative intensity between A^0 and A^- peaks. Specifically, interfacial charge transfer results in net charge carriers (electron or hole) accumulating in WS_2, which may combine with the neutral excitons and become charge trions (Supplementary Figure 3(d)), causing a PB feature at the trion resonance due to band filling effect. In comparison, energy transfer leaves neutral excitons in WS_2, resulting a PB peak at the exciton resonance (Supplementary Figure 3(e)). Base on this assumption, charge transfer is found to dominate the interfacial transfer process in WS_2-G heterostructure (Supplementary Figure 3(c)), prevailing over the energy transfer. Unfortunately, only TA spectrum is not enough to further distinguish whether the hole or electron transfer occurs in this heterostructure, both of which have been discussed in previous reports[4, 7].

We also use a series of sub-bandgap photon energy, ranging from 1.77 to 0.48 eV, to pump the sample, in which the carrier transfer direction is from graphene to WS_2. Supplementary Figure 4(a) presents the TA amplitude per unit pump fluence (μJ cm^{-2}) versus excitation energy from different sample regions. For comparison, the up-bandgap pumping signal (2.1 eV) is also plotted. As the pump energy drops below the WS_2 bandgap, excitons in the controlled monolayer WS_2 immediately decrease to an undetectable level, indicating no two-photon absorption in our pump fluence level. In contrast, a considerable, spectrally flat TA response is found in G-WS_2 and G-*h*-BN-WS_2 under the sub-bandgap pumping, even when the pump energy drops to as low as 0.48 eV (2600 nm). This

is a natural result given the uniform absorption of graphene in a very broad band from terahertz (THz) to ultraviolet (UV)[8]. The same spectrally flat signal was also found in the photocurrent response in the G-WSe_2-G device[9].

We note that in this sub-bandgap pumping case, energy and charge transfer can also be distinguished from the spectrally resolved TA response, as shown in Supplementary Figure 4(b). Charge transfer again prevails over the energy transfer in G-WS_2 region, which may be beneficial for graphene-based broadband light conversion or detection devices such as photodetectors or solar cells[10, 11]. The more interesting issue is found in G-*h*-BN-WS_2 region (Supplementary Figure 4(c) and Supplementary Figure 5), which also exhibits a considerable response from the neutral exciton. We have shown above that the 4-5 layer *h*-BN spacer is thick enough to block the short-range (1 nm) interfacial charge transfer[4], but it cannot completely suppress the relatively long range (up to several nm) Förtser-type energy transfer[12]. Thus, the neutral exciton response in G-*h*-BN-WS_2 region is attributed to this long range Förtser-type energy transfer.

Supplementary Figure 4(c) shows a comparison of the carrier relaxation dynamics induced by the charge and energy transfers. The excitons decay very fast in both cases, with ~1.3 (2) ps for charge (energy) transfer (see inset of Supplementary Figure 5 for other pump energy). This indicates that ultrafast nonradiative recombination in WS_2 dominates the carrier relaxation process after the interfacial carrier transfer[2].

Supplementary Note 3: Interfacial thermal transport model

To simulate the dynamics of WS_2 lattice temperature rise, we consider the energy relaxation pathways shown in Supplementary Figure 6. Upon sub-bandgap excitation, the photo energy is transferred to graphene carriers via interband or intraband transition. These initial carriers are non-equilibrium, and will share their energy to other low-energy carrier through carrier-carrier collisions in the following ~30 fs, establishing a quasi-equilibrium hot carrier distribution with a defined temperature of T_1. T_1 can be estimated from the equation of $T_1 = \sqrt{T_0^2 + 2P_{\mathrm{in}} / \gamma}$ (see in the main text), where T_0 is the ambient temperature, $\gamma = \frac{2\pi\mu k_{\mathrm{B}}^2}{3\hbar^2 v_{\mathrm{F}}^2}$ is the 2D Sommerfeld constant, with graphene Fermi energy of $\mu = 0.4\ \mathrm{eV}$ (considering the impurity/defect doping of the heterostructures) and Fermi velocity of $v_{\mathrm{F}} = 1\times10^6\ \mathrm{m/s}$, and $P_{\mathrm{in}} = \alpha P \eta_{\mathrm{heat}}$ is the pump fluence that is absorbed by graphene and transferred to the electron bath. Here, $\alpha = 5\%$ is the absorption coefficient of graphene on SiO_2/Si substrate[13], P is the incident pump fluence, and $\eta_{\mathrm{heat}} = 70\%$ is transfer efficiency[9].

After the ultrafast excitation and thermalization, the build-up hot carriers lose their energy mainly through two pathways: one is that a small fraction of hot carrier may transfer through the G-WS_2 interface, causing electronic excitation of WS_2 in the first 10 ps. Although this pathway leads to a considerable TA signal, it is still negligible compared to the second pathway, phonon emission. Most of the hot carriers in graphene deliver their energy to the phonon bath via the ultrafast (~1 ps) phonon emission. Various phonon cooling processes have been proposed, including intrinsic acoustic phonons[14], interaction with remote surface polar phonon modes[15], and disorder-enhanced supercollisions with acoustic phonons [16-18]. As discussed in the main text, we employ the supercollision model, with an energy loss rate of $H_{\mathrm{e-L}} = A\left(T_{\mathrm{e-G}}^3 - T_{\mathrm{L-G}}^3\right)$, where $A = 5.5\times10^8\gamma$ is

the rate coefficient, $T_{\mathrm{e-G}}\,(T_{\mathrm{L-G}})$ is the graphene carrier (lattice) temperature. The following energy relaxation processes consist of thermal transport from graphene to WS_2 lattice and then WS_2 lattice to SiO_2/Si substrate, which are limited by the thermal conductance of the G-WS_2 interface ($\Gamma_{\mathrm{G-WS_2}}$) and WS_2/SiO_2 interface (Γ_0) respectively. Base on these pathways, we have:

$$\begin{aligned}
C_{\mathrm{e-G}}\frac{\partial T_{\mathrm{e-G}}}{\partial t} &= -A\left(T_{\mathrm{e-G}}^3 - T_{\mathrm{L-G}}^3\right)\\
C_{\mathrm{L-G}}\frac{\partial T_{\mathrm{L-G}}}{\partial t} &= A\left(T_{\mathrm{e-G}}^3 - T_{\mathrm{L-G}}^3\right) - \Gamma_{\mathrm{G-WS_2}}\left(T_{\mathrm{L-G}} - T_{\mathrm{WS_2}}\right)\\
C_{\mathrm{L-WS_2}}\frac{\partial T_{\mathrm{L-WS_2}}}{\partial t} &= \Gamma_{\mathrm{G-WS_2}}\left(T_{\mathrm{L-G}} - T_{\mathrm{L-WS_2}}\right) - \Gamma_0\left(T_{\mathrm{L-WS_2}} - T_0\right)
\end{aligned} \tag{1}$$

where $C_{\mathrm{e-G}}=\gamma T_{\mathrm{e-G}}$ is the graphene electronic heat capacity, $C_{\mathrm{L-G}} = 1.53\times10^{-6}T_{\mathrm{L-G}}$ J m^{-2}K^{-1} is the graphene lattice heat capacity[19, 20], $C_{\mathrm{L-WS_2}} = 4.5\times10^{-4}$ J m^{-2}K^{-1} is the WS_2 lattice heat capacity[21] and $T_0 = 300$ K is the ambient temperature.

When *h*-BN spacer is inserted into G-WS_2 heterostructure, the graphene lattice energy is first transported to *h*-BN layer, then to the WS_2, and finally dissipates into SiO_2/Si substrate. The above equations are changed as:

$$\begin{aligned}
C_{\mathrm{e-G}}\frac{\partial T_{\mathrm{e-G}}}{\partial t} &= -A\left(T_{\mathrm{e-G}}^3 - T_{\mathrm{L-G}}^3\right)\\
C_{\mathrm{L-G}}\frac{\partial T_{\mathrm{L-G}}}{\partial t} &= A\left(T_{\mathrm{e-G}}^3 - T_{\mathrm{L-G}}^3\right) - \Gamma_{\mathrm{G-BN}}\left(T_{\mathrm{L-G}} - T_{\mathrm{BN}}\right)\\
C_{\mathrm{BN}}\frac{\partial T_{\mathrm{BN}}}{\partial t} &= \Gamma_{\mathrm{G-BN}}\left(T_{\mathrm{L-G}} - T_{\mathrm{BN}}\right) - \Gamma_{\mathrm{BN-WS_2}}\left(T_{\mathrm{BN}} - T_{\mathrm{L-WS_2}}\right)\\
C_{\mathrm{L-WS_2}}\frac{\partial T_{\mathrm{L-WS_2}}}{\partial t} &= \Gamma_{\mathrm{BN-WS_2}}\left(T_{\mathrm{BN}} - T_{\mathrm{L-WS_2}}\right) - \Gamma_0\left(T_{\mathrm{L-WS_2}} - T_0\right)
\end{aligned} \tag{2}$$

where $C_{\mathrm{BN}} = 1.4\times10^{-5}T_{\mathrm{BN}}$ J m^{-2}K^{-1} is the heat capacity of a 4 layer *h*-BN[20], T_{BN} is the *h*-BN lattice temperature, $\Gamma_{\mathrm{G-BN}} = 52$ MW m^{-2} K^{-1} is the thermal conductance at the G/*h*-BN interface[22], $\Gamma_{\mathrm{BN-WS_2}}$ is the thermal conductance at the *h*-BN/WS_2 interface.

The kinetics of the WS_2 lattice temperature rise is governed by the relative thermal conductance of $\Gamma_{\mathrm{G-WS_2}}$ and Γ_0 ($\Gamma_{\mathrm{BN-WS_2}}$ and Γ_0) in G-WS_2 (G-*h*-BN-WS_2) heterostructure. Our thermal

transport model predicts the values of $\Gamma_{G-WS_2} \sim 5 \pm 2$ MW m^{-2} K^{-1} and $\Gamma_{BN-WS_2} \sim 4 \pm 2$ MW m^{-2} K^{-1}. These values, although 3~4 times smaller than that in *h*-BN-MoS_2 interface[22], are within the experimental errors, since the contact thermal conductance is closely related to the coupling strength and the impurity/defect at the interface. The decay rate of the TA signal after the APR peaking time (t_{APR}, Supplementary Figure 7) is governed by the dissipative rate (Γ_0) of the WS_2 thermal energy, which is estimated to be $1.0 \pm 0.3\,(0.8 \pm 0.2)$ MW m^{-2} K^{-1} in G-WS_2 (G-*h*-BN-WS_2) region. This low thermal conductance may be attributed to the low out-plane thermal conductivity of the WS_2[23] or to the bad interfacial thermal contact at WS_2/ SiO_2 interface. Regardless of this, the slow energy dissipative rate here can efficiently confine the thermal energy into WS_2 for carrier re-excitation, and of course, at the cost of slower response time of the device.

Supplementary Note 4: Discussion of the TA spectrum caused by the lattice heating effect.

Upon sub-bandgap pumping, the excited carriers in graphene transfer most of their energy to the graphene lattice via phonon emission and then to the WS_2 lattice via interfacial thermal transport. The heating of the WS_2 lattice leads to a red shift of the exciton resonance (optical bandgap) due to the electron-phonon coupling. This red shift of exciton resonance may induce an antisymmetric derivative TA spectrum in pump-probe experiment. The lattice heat induced derivative TA spectrum seems very similar to the one we acquired at the delay of > 100 ps (Supplementary Figure 7), which however, is attributed to the phononic excitation in the main text. To distinguish the phononic excitation and the crystal heating induced band red shift, we now provide an in-depth discussion.

To begin with, a roughly quantitative estimation of the pure crystal heating and the phononic excitation is performed, based on the experimental TA spectrum. For a monolayer WS_2 on a Si/SiO_2 substrate, the reflective spectrum caused by the band edge exciton (A^0 or A^-) absorption follows a Lorentzian line shape:

$$R_0(E) = 1 - \frac{A_0}{1 + 4\left(\frac{E - E_0}{\sigma_0}\right)^2}$$

Where $A_0 \approx 0.1$ is the absorptivity of the monolayer WS_2, E_0 is the optical resonance whose value varies from 1.98 to 2.02 eV depending on the optical excitation species (A^0 or A^-), $\sigma_0 \approx 40$ meV is the line width of the reflective spectrum (i.e. full width at half maximum). Here, for simplicity, the contributions from free carrier absorption continuum and Rayleigh scattering are neglected as they have little influence on TA signal. When the monolayer WS_2 is excited by the pump pulse, all these parameters change accordingly. Specifically, phononic excitation induces a band bleaching, with $\Delta A = A - A_0 < 0$, and both of the phononic excitation and crystal heating

cause a band shift $\Delta E = E - E_0$ and a spectral broadening $\Delta\sigma = \sigma - \sigma_0$. Considering all these factors, the antisymmetric TA signal cannot provide too much information, as shown in Supplementary Figure 7. To be more specific, an antisymmetric TA spectrum is naturally acquired if we merely consider the crystal heating induced band shift ΔE (blue lines, Supplementary Figure 7). The fitting value of the band shift is around 1.8 ~ 2.3 eV, corresponding to a lattice temperature increase of 6 ~ 8 K when considering a band shift rate of ~0.3 eV K^{-1} [24]. This is consistent well with the estimated value of ~11 K from the thermal transport model provided in note 3. However, when including a strong bleaching effect of $\Delta A = -4 \sim -5\%$ and a slight broadening of $\Delta\sigma = 2.5 \sim 5$ meV, the antisymmetric characteristics of the TA spectrum are well preserved (red dash, Supplementary Figure 7). Note that while the ΔE and the $\Delta\sigma$ can be caused by both the phononic excitation and crystal heating (see below for detail), the ΔA can only be induced by the phononic excitation. Thus, it is not sufficient to distinguish these two effects simply from the TA spectrum, even when it appears to be 'antisymmetric'. An actual G-WS_2 device for photocurrent extraction is required to completely distinguish them, since crystal heating cannot generate photocarriers.

Although the phononic excitation and crystal heating effects cannot be completely distinguished, the systematic experimental data from different heterostructures indicate that the phononic excitation should play an important role for the slow TA feature. Supplementary Figure 8 shows a comparison of the TA response (both fast and slow components) from 3 different samples. The shapes of the spectrum are ever-changing whether they are measured at short (~1 ps) or long (~100 ps) delay times. The slow TA spectrum at the third sample is even completely asymmetric, with a PB peak much stronger than the PIA one (Supplementary Figure 8f). This indicates that at least in

Sample 3, phononic excitation should play a dominant role for the slow TA response, considering that simply crystal heating cannot induced a net band filling effect.

Another indirect experimental result showing that phononic excitation plays a role is found in G-MoS_2 sample, in which the monolayer MoS_2 is nearly intrinsic doped and thus the phononic excitation is seriously suppressed, as discussed in Section 8. In this case, the initial electronic excitation induced by interlayer carrier transfer still exists, indicating good interface quality between the two monolayers. In principle, if the crystal heating effect makes a contribution, such a good interface coupling can support an effective interlayer thermal transport and induce a significant antisymmetric TA response at long delay time. However, no secondly-rise antisymmetric TA signal is observed in our measurement, indicating that crystal heating might be negligible for the TA response of the G-MoS_2 sample at our pumping fluence level.

After confirming the existence of phononic excitation, we now have a brief discussion on the origin of the PIA and PB features in the TA spectrum of TMDCs family from the perspective of many-body interaction. The PB peak is relatively simple, which often occurs at the excitonic resonance and is mainly attributed to the band filling effect of the band edge excitons. While the PIA feature is distinct from sample to sample, both in peak amplitude and peak energy. As shown in Supplementary Figure 8, the PIA amplitude can be either smaller than or comparable to the PB peak, and it may locate at an energy smaller or/and higher than the PB feature. Furthermore, the TA spectra in the same sample but different excitation conditions (i.e. electronic or phononic excitation) are also distinct.

Such variable PIA feature is commonly attributed to many-body effect in TMDCs. Specifically, the presence of the pump-excited carriers strongly alters the optical response of the latter-coming

probe pulse. Several physical processes contribute to this absorption change: i). Phase-space filling (or Pauli-blocking) and Coulomb scattering of the carrier result in a decrease oscillator strength and spectral broadening, as elucidated in Supplementary Figure 9(a). In TA spectrum, these two effects lead to a PB peak at the exciton resonance and simultaneously a small PIA feature at the energy nearby. ii). Screening of the Coulomb attraction results in a reduced exciton binding energy and thus a blue shift of the A exciton resonance, as shown in Supplementary Figure 9(b). This effect induces a PB peak at the primary band and a PIA peak at the newly blue-shift band. iii). Screening of the Coulomb repulsion causes a reduced quasi-particle bandgap (or electronic bandgap) and thus a red shift of the A exciton resonance, resulting in a PB feature and a red-shift PIA feature, as shown in Supplementary Figure 9(c). Note that the last two mechanisms have opposite effect on the A exciton resonance, leading to a band shift either to higher or lower energy. Moreover, these two processes partially compensate each other and give rise to an overall peak shift no more than a few tens of millielectronvolts. The shift direction as well as the shift amplitude depends on a variety of material properties and excitation conditions[24], including the effective temperature and density of the photoexcited carriers, as well as the ratio of the excitons to free electrons and holes. Therefore, with the impact of the complicated many-body effect, it is no surprise to find that different WS_2 samples show different TA spectra, and even the same sample exhibits different TA spectra under different exciting conditions, e.g. electronic and phononic excitations.

Supplementary Note 5: Interfacial coupling-dependent APR effect

The APR effect is essentially based on the interfacial thermal transport, for which the interlayer thermal conductance (Γ) is closely related to the interface contact. Thus it is necessary to explore the dependence of the interface coupling strength on the APR efficiency, as there is no ideal interface in practical devices.

To acquire G-WS_2 heterostructures containing regions with significantly different coupling strength, we prepare new samples using the dry transfer technique similar to the case of the main text but without the final annealing process. The absence of the annealing process remains some local strains at the interface generated during the transfer process, leading to an inhomogeneous interface containing both well and terrible coupling strengths. A typical non-annealed G-WS_2 sample is shown in Supplementary Figure 10(a), whose interface quality is characterized via PL quenching image under up-bandgap excitation (532 nm). As shown in Supplementary Figure 10(b), regions with both large (well interlayer coupling) and negligible (terrible interlayer coupling) PL quenching are found in the G-WS_2 heterstructures, providing an ideal platform to study the coupling-dependent APR effect. Again, we use a femtosecond pulse with a sub-bandgap photon energy of 0.48 eV to pump the graphene and trace the carrier density excited in WS_2. Since the electronic and phononic excitations dominate the excited carriers at different time scales, they can be separately characterized simply by changing the time delays. Supplementary Figure 10(c) show the TA intensity images induced by the electronic (0.5 ps delay, up panel) and phononic (100 ps delay, below panel) excitations. Both the TA maps show the same profile as that of PL intensity, indicating that both the two excitations are closely related to the interface quality. Specifically, regions with stronger coupling strength (weaker PL intensity) always show more efficient electronic

and phononic excitations. The positive correlation between phononic excitation and interfacial coupling strength can also be predicted from our APR model. An improvement of coupling strength may induce a rise of the interfacial thermal conductance between G and WS_2 layers. In this case, if heat dissipated rate at WS_2-SiO_2/Si interface does not change, the maximum thermal energy stored in WS_2 at a particular time delay (t_{APR}) will increase, leading to the increase of the excited carrier density.

For further quantitative study, we choose 3 junctions (Area 1, 2 and 3, marked in Supplementary Figure 10(b)) where the coupling strength gradually increases for TA kinetics measurement, with the TA spectrum relaxations showing in Supplementary Figure 10(d). Both the electronic and phononic excitations are clearly found in the 3 areas, with different time scales of < 10 ps and > 10 ps, respectively. For comparison, we plot the TA spectra of the 3 areas together, as shown in Supplementary Figure 10(e). Although the electronic and phononic excitations are both positively related to the interface coupling strength, the former is much more sensitive. With coupling strength increasing from area 1 to 3, the electronic excitation increases ~13 times, while the phonon excitation only enhances twice. This can be attributed to different effective transfer ranges of the electron and phonon. Carrier transfer is a short-range process that relies on the orbital overlap between two adjacent layers (~1 nm) or on near-field dipole-dipole coupling (up to several nm), which is extremely sensitive to the interlayer distance. While the phonon transport relies on collective vibration of the crystal lattice, whose effective range is much longer than that of carrier transfer.

Supplementary Note 6: Robust stacking-independent APR effect

Interfacial transfer process between graphene and WS_2 involves the variation of parallel momentum vector and band alignment when their twist angle changes. General beliefs and theoretical studies thus have shown that the interfacial charge transfer processes, including both the rate and efficiency, depend sensitively on the twist angle (φ) between two adjacent layers. Thus, it is very important and urgent to study the effect of the twist angle and energy band misalignment on the APR process in G-WS_2 heterostructures, not only for understanding the fundamental physics, but also for optimizing applications of 2D heterostructures.

The twist angle φ is defined as the angle between the zigzag (or armchair) directions of the G and WS_2 layers, where φ can vary from 0-30° considering the 6-fold symmetry of the G lattice. To eliminate the diversity from sample to sample, we choose the monolayer WS_2 layer grown by chemical vapor deposition (CVD) rather than mechanical exfoliation, which contains WS_2 triangles with different crystal orientations in one sample. Then a larger mechanical exfoliated graphene flake was transferred onto the CVD WS_2 film, forming heterostructures with different twist angles. The orientation of graphene is determined by Raman spectroscopy, while the orientation of WS_2 triangle is identified from the optical image, with three sides orienting at zigzag direction.

A typical G-WS_2 heterostructure on SiO_2/Si substrate is shown in Supplementary Figure 11(a). A femtosecond laser pulse (0.48 eV, ~34 μJ cm^{-2}) is used to excite the heterostructure to study the APR process, with a typical carrier relaxation kinetics (φ = 1.5°) showing in Supplementary Figure 11(b). Although the secondly rise peak is not so obvious from naked eyes compared to the samples used in the main text, one can still see a nearly flat platform from 20 to ~100 ps, which is caused by the APR effect. Next, to determine the dependence of phononic excitation on angular alignment, we

carry out TA intensity mapping at two fixed delay time, 0.5 ps (electronic excitation) and 100 ps (phononic excitation), as shown in Supplementary Figure 11(c) and (d). Three heterostructure configurations are shown in the two images, with φ = 1.5°, 23° and 30°. Within the range of noise, both the electronic and phononic excitation intensities show no correlation with the twist angle. The TA spectra caused by these two excitations are also independent on the angular alignment (Supplementary Figure 11(e)). To further give a quantitative comparison, we made a statistic on these two excitations from 11 configurations in this sample, with data of each configuration averaging from 10 different regions. As shown in Supplementary Figure 11(f), again, there is no obvious dependence between electronic (phononic) excitation and twist angle.

The absence of correlation between electronic (phononic) excitation and φ seems quite counterintuitive, and is also not consistent with some previous theory studies[25]. However, we note that many experimental studies also show similar result in different types of TMDC heterostructures, including MoS_2-WSe_2[26], WS_2-MoS_2[27, 28] and MoS_2-$MoSe_2$[29]. The interlayer charge transfer is found to be extremely robust against varying interlayer twist angles. Two mechanisms are proposed accounting for this robust carrier transfer. The first one is hot carrier transfer[26], namely, the excess kinetic energy of the transferred carriers allows them to sample a broad range of K space above the CBM (K point). The second mechanism is the coexistence of various local stacking configurations caused by atomically local structural inhomogeneity during the sample preparation[28]. Since the lattice constants of the graphene (2.46 Å) and WS_2 (3.15 Å) are different, massive local strains are generated when the two layers match together and further amplified in the following annealing process. In conjunction with the different thermal expansion coefficients, the local strains may finally result in interlayer sliding, providing different local stacking configurations for carrier

transfer.

Although these two mechanisms are proposed for carrier transfer, i.e. electronic excitation, similar concept can be applied to the phononic excitation. The existence of hot phonon and the heterogeneous interlayer stretching/sliding may also induce a robust phononic excitation against varying twist angle between the two monolayer components.

Supplementary Note 7: APR process in WS_2 with different atomic layers

WS_2 with different atomic layers affect the APR process through changing of the heat capacity, which is proportional to the number of layers, i.e., $C_{\mathrm{WS_2(n)}} = nC_{\mathrm{WS_2(1)}}$, where $C_{\mathrm{WS_2(1)}} = 4.5\times10^{-4}\ \mathrm{J\ m^{-2}\ K^{-1}}$ is the monolayer heat capacity[21]. According to our thermal transport model, the increase of $C_{\mathrm{WS_2}}$ leads to the decrease of lattice temperature rise $\Delta T_{\mathrm{L-WS_2}}$ and thus the APR intensity, as the total energy stored in the G-WS_2 system is constant under a fixed pump fluence. Furthermore, the increase of WS_2 layer will significantly increase the peak delay time of APR-induced photo carriers (t_{APR}) because it takes more time for thermal transport and balance. We can simulate the correlation between $\Delta T_{\mathrm{L-WS_2}}$ (t_{APR}) and the WS_2 layers (n) based on our thermal transport model, as shown in Supplementary Figure 12a(b).

Next, experiment is carried out to confirm our model, with G-WS_2 heterostructure prepared from mechanically exfoliated graphene and WS_2, as shown in Supplementary Figure 12(c). Note that in this heterostrcture different layers of WS_2 are originated from only one bulk single crystal, ensuring the consistency of the doping level (E_{F}) at different layers, which is also closely related to the APR efficiency. 2D pseudo-color TA maps of different heterostructure regions, including G-1L WS_2, G-few layer WS_2 and G-bulk WS_2, are shown in Supplementary Figure 12(d), with a pump energy of 0.48 eV. Although not as obvious as the sample used in the main text, all the 3 regions show slightly APR effect, with characteristic time (t_{APR}) showing by the vertical dashed line. These experimental t_{APR} are compared to the simulated results in Supplementary Figure 12(b). The excellent agreement between the experimental and simulated results again confirms the APR process. Note that with increasing WS_2 layers, the relaxation of the carriers (the PB peak) caused by the initial electronic excitation is greatly slowed down, which may seriously hinder the observation of the following slow

APR effect. Nevertheless, from the kinetics of the PIA peaks (~2.0 eV for few layer WS_2 and ~1.95 eV for bulk WS_2), we can still find the secondly rise feature resulting from the APR process.

Supplementary Note 8: APR process in atomic MoS_2.

A new heterostructure, G-MoS_2, was prepared to show the possible APR process, as shown in Supplementary Figure 13(a). Unfortunately, no APR effect was found in this as-prepared sample under pumping with 0.48 eV photon energy. As shown in Supplementary Figure 13(b-c), the carrier density increases quickly in the first 0.5 ps, which is caused by the interfacial carrier transfer rather than the APR process. Then in the following delay time, the carriers decrease monotonously throughout the whole detection range (0~1.4 ns). The shape of the TA spectrum excited by 0.48 eV light pulse is also the same at all delay time (Supplementary Figure 13(d)), further indicating that interfacial carrier transfer is the only mechanism accounting for the excited carriers in MoS_2.

The absence of APR process in this sample is attributed to the nearly intrinsic nature of the MoS_2 layer. As shown in Supplementary Figure 13(e), the TA spectra are completely the same in both controlled MoS_2 and heterostructure regions, all dominated by the neutral excitons (A^0 and B^0). This indicates that E_F of MoS_2 locates at an energy level near the neutral point, far from the conduction band minimum (E_c). According to our model (Equation 1 in the main text), the APR efficiency is exponentially dependent on $E_c - E_F$. Thus, the nearly intrinsic nature of MoS_2 may induce a negligible APR effect. In stark contrast, the optical response of WS_2 in the heterostructure region is completely dominated by the charge trion (Figure 2(d) in the main text), leading to the dramatic enhancement of the APR efficiency.

Note that although APR process is not found in the as-prepared G-MoS_2 heterostructure, if we can raise the MoS_2 Fermi level from an intrinsic state to an energy level near the E_c, just as the case of G-WS_2, the APR efficiency will also be significantly enhanced. Actually, the APR efficiency is also distinct in different G-WS_2 samples, possibly caused by the change of Fermi energy. In the

main text we have a simple estimate of the influence of E_F on the detection signal $\Delta R / R_0$, a 100 meV rise of Fermi energy may lead to a dramatic increase of $\Delta R / R_0$ from 0.001 to 0.027. Thus, electrical gate control may be an efficient strategy to improve the APR efficiency.

Supplementary References:

1. He, J. *et al.* Electron transfer and coupling in graphene-tungsten disulfide van der Waals heterostructures. *Nat. Commun.* **5,** 5622 (2014).
2. Yuan, L. *et al.* Photocarrier generation from interlayer charge-transfer transitions in WS_2-graphene heterostructures. *Sci. Adv.* **4,** e1700324 (2018).
3. Hill, H. M., Rigosi, A. F., Raja, A., Chernikov, A. & Heinz, T. F. Exciton broadening in WS_2/graphene heterostructures. *Phys. Rev. B* **96,** 205401 (2017).
4. Lorchat, E., Berciaud, S. & Froehlicher, G. Charge versus energy transfer in atomically thin graphene-transition metal dichalcogenide Van der Waals heterostructures. *Phys. Rev. X* **8,** 011007 (2018).
5. Nie, Z. *et al.* Ultrafast carrier thermalization and cooling dynamics in few-layer MoS_2. *ACS Nano* **8,** 10931-10940 (2014).
6. Plechinger, G. *et al.* Trion fine structure and coupled spin-valley dynamics in monolayer tungsten disulfide. *Nat. Commun.* **7,** 12715 (2016).
7. Aeschlimann, S., Rossi, A., Chávez-Cervantes, M., Krause, R. & Gierz, I. Direct evidence for efficient ultrafast charge separation in epitaxial WS_2/graphene heterostructures. *Sci. Adv.* **6,** eaay0761 (2020).
8. Bonaccorso, F., Sun, Z., Hasan, T. & Ferrari, A. C. Graphene photonics and optoelectronics. *Nat. Photon.* **4,** 611-622 (2010).
9. Massicotte, M. *et al.* Photo-thermionic effect in vertical graphene heterostructures. *Nat. Commun.* **7,** 12174 (2016).
10. Georgiou, T. *et al.* Vertical field-effect transistor based on graphene-WS_2 heterostructures for flexible

and transparent electronics. *Nat. Nanotechnol.* **8,** 100-103 (2012).

11. Britnell, L. *et al.* Strong light-matter interactions in heterostructures of atomically thin films. *Science* **340,** 1311-1314 (2013).

12. Xu, W. *et al.* Determining the optimized interlayer separation distance in vertical stacked 2D WS_2:hBN:MoS_2 heterostructures for exciton energy transfer. *Small* **14,** 1703727 (2018).

13. Stauber, T., Peres, N. M. R. & Geim, A. K. Optical conductivity of graphene in the visible region of the spectrum. *Phys. Rev. B* **78,** 085432 (2008).

14. Bistritzer, R. & Macdonald, A. H. Electronic cooling in graphene. *Phys. Rev. Lett.* **102,** 206410 (2009).

15. Low, T., Perebeinos, V., Kim, R., Freitag, M. & Avouris, P. Cooling of photoexcited carriers in graphene by internal and substrate phonons. *Phys. Rev. B* **86,** 045413 (2012).

16. Graham, M. W., Shi, S. F., Ralph, D. C., Park, J. & Mceuen, P. L. Photocurrent measurements of supercollision cooling in graphene. *Nat. Phys.* **9,** 103-108 (2013).

17. Betz, A. C. *et al.* Supercollision cooling in undoped graphene. *Nat. Phys.* **9,** 109 (2012).

18. Song, J. C. W., Reizer, M. Y. & Levitov, L. S. Disorder-assisted electron-phonon scattering and cooling pathways in graphene. *Phys. Rev. Lett.* **109,** 106602 (2012).

19. Meng, S., Schelling, P. K. & Keblinski, P. Heat transfer mechanism across few-layer graphene by molecular dynamics. *Phys. Rev. B* **88,** 045444 (2013).

20. Tohei, T., Kuwabara, A., Oba, F. & Tanaka, I. Debye temperature and stiffness of carbon and boron nitride polymorphs from first principles calculations. *Phys. Rev. B* **73,** 064304 (2006).

21. Sie, E. J. *et al.* Observation of exciton redshift-blueshift crossover in monolayer WS_2. *Nano Lett.* **76,** 181 (2017).

22. Liu, Y. *et al.* Thermal conductance of the 2D MoS_2/*h*-BN and graphene/*h*-BN Interfaces. *Sci. Rep.* **7,** 43886 (2017).

23. Jiang, P., Qian, X., Gu, X. & Yang, R. Probing anisotropic thermal conductivity of transition metal dichalcogenides MX_2 (M = Mo, W and X = S, Se) using time-domain thermoreflectance. *Adv. Mater.* **29,** 1701068 (2017).

24. Ruppert, C., Chernikov, A., Hill, H. M., Rigosi, A. F. & Heinz, T. F. The role of electronic and phononic excitation in the optical response of monolayer WS_2 after ultrafast excitation. *Nano Lett.* **17,** 644-651 (2017).

25. Zhang, J. *et al.* Interlayer-state-coupling dependent ultrafast charge transfer in MoS_2/WS_2 bilayers. *Adv. Sci.* **4,** 1700086 (2017).

26. Zhu, H. *et al.* Interfacial charge transfer circumventing momentum mismatch at two-dimensional van der Waals heterojunctions. *Nano Lett.* **17,** 3591-3598 (2017).

27. Hong, X. *et al.* Ultrafast charge transfer in atomically thin MoS_2/WS_2 heterostructures. *Nat. Nanotechnol.* **9,** 682-686 (2014).

28. Ji, Z. *et al.* Robust stacking-independent ultrafast charge transfer in MoS_2/WS_2 bilayers. *ACS Nano* **11,** 12020-12026 (2017).

29. Ceballos, F., Bellus, M. Z., Chiu, H. & Zhao, H. Ultrafast charge separation and indirect exciton formation in a MoS_2–$MoSe_2$ van der Waals heterostructure. *ACS Nano* **8,** 12717-12724 (2014).
